# A 3D evaluation of dyssynchrony may offer an advantage over a 2D approach; a cardiovascular MRI method for dyssynchrony quantification

**DOI:** 10.1186/1532-429X-11-S1-P9

**Published:** 2009-01-28

**Authors:** Robert WW Biederman, Frank Grothues, Helmut Klein, Ronald B Williams, June A Yamrozik, Geetha Rayarao, Diane A Vido, Christof Huth, Mark Doyle

**Affiliations:** 1 Allegheny General Hospital, The Gerald McGinnis Cardiovascular Institute, Pittsburgh, PA USA; 2 University Hospital Magdeburg, Magdeburg, Germany

**Keywords:** Optimal Medical Therapy, Tissue Doppler Echocardiography, Standard Echocardiography, Dyssynchrony Index, Post Treatment Effect

## Introduction

Detection of dyssynchrony is primarily performed by EKG and/or tissue Doppler echocardiography, sampling the heart in selected basal segments. Limited approaches for dyssynchrony have been performed by cardiovascular MRI (CMR).

## Hypothesis

We hypothesize that 3D global assessment of dyssynchrony may be more sensitive than conventional regional 2D analysis.

## Methods

At baseline, 8 patients (47 ± 9 yrs) with mean NYHA Class 2.3 ± 0.5 on optimal medical therapy (maintained throughout the study) underwent 3D CMR (1.5 T GE) to assess LV function. Using Medis Mass software (Leiden, The Netherlands), endo and epicardial boundaries were outlined in multiple contiguous short-axis slices. For each slice the myocardium was circumferentially divided into 16 equally spaced segments. End-systolic (ES) time was automatically identified as time of maximal wall thickening and the dyssynchrony index taken as the dispersion of ES times. Patients underwent HeartNetTM (Paracor Medical Inc, Sunnyvale, CA) placement. A follow-up CMR was performed at 6 months. In total >800 data points were generated for each heart (as compared to 6 by standard echocardiography). The dispersion of the ES time was analyzed for pre to post treatment effect for the basal region separately using all measured points and for 4, 6, or 8 equally spaced circumferential regions, each set starting at several offset values, resulting in 11 regional data sets.

## Results

All patients survived HeartNet™ placement and were available for 6 month follow-up. When using all segments to describe the dispersion of ES time pre to post, a statistically significant change in the dyssynchrony index was observed (254 vs. 220 ms, p < 0.001). When assessed using the separate 2D analysis, a pre-post change in dyssynchrony was only detected in 3 out of 11 data series (36%).

## Conclusion

By its nature, dyssynchrony is a heterogeneous phenomenon. When assessed using a 3D CMR approach, a pre to post treatment effect was detectable. However, when restricting measurements to 4, 6, or 8 equally spaced regions (analagous to a 2D echocardiographic approach), the chance of detecting the change in dyssynchrony substantially dropped, indicating that the phenomenon of Dyssynchrony should be assessed using a global 3D as opposed to regional 2D approach, *irrespective* of assessment modality.

